# A new benchmark for research integrity: innovation in the artificial intelligence era and safeguarding the reliability of the scholarly research published in *Women’s Health Nursing*

**DOI:** 10.4069/whn.2026.03.16

**Published:** 2026-03-31

**Authors:** Ju-Hee Nho

**Affiliations:** College of Nursing, Research Institute of Nursing Science, Jeonbuk National University, Jeonju, Korea

The integrity of the scholarly record underlies clinical decision-making, policy development, and scientific progress [1]. In recent years, research and scholarly publishing have undergone rapid transformation. One of the most influential developments has been the emergence of generative artificial intelligence (AI), which has the potential to substantially improve research productivity and efficiency [2]. At the same time, however, these technological advances have created new challenges to research integrity, including the spread of paper mills and increasingly sophisticated forms of data manipulation [3].

Whereas research integrity was once maintained largely through mutual trust and retrospective responses to misconduct, the current era requires systematic and proactive safeguards [4]. In response to these changes, *Women’s Health Nursing* will actively embrace the innovative potential of AI technologies while reinforcing its efforts to ensure the reliability and credibility of submitted manuscripts.

## Adherence to the International Association of Scientific, Technical and Medical Publishers nine-category framework for AI activities and enhancement of transparency

To promote global standards in publishing ethics, the International Association of Scientific, Technical and Medical Publishers (STM) has proposed a nine-category framework for AI-related activities in scholarly publishing [5]. This framework provides an important mechanism for reducing uncertainty and improving transparency for authors, reviewers, and readers. The nine categories are outlined below. They include language refinement, translation, content generation, illustrative image generation, data and code refinement, visualization, and assistance with reference identification. However, presenting AI-generated content as though it were original research data derived from non-machine sources constitutes serious research misconduct and is strictly prohibited [5].

The nine STM categories of AI use are as follows [5]:

1) Language refinement: activities in which AI tools are used to suggest linguistic improvements that enhance the clarity and readability of text written by humans.

2) Translation: the use of AI tools to assist in translating an author’s original work into another language for publication.

3) Content generation: the use of AI to generate part or all of the manuscript text.

4) Illustrative image generation: the generation of images solely for aesthetic or explanatory purposes, without representing actual research data.

5) Data refinement and formatting: the use of AI to improve the presentation or formatting of research data reported in the manuscript.

6) Data visualization: the generation or refinement of visual representations of research data or results.

7) Code refinement: the use of AI tools to improve the readability and clarity of research code without altering its functionality.

8) Reference assistance: AI-supported identification or recommendation of relevant literature for inclusion in the manuscript’s reference list.

9) Deceptive AI content (prohibited): the presentation of AI-generated content as though it were original research data obtained from non-machine sources.

## Disclosure requirements and transparency policy

Authors must clearly disclose whether AI tools were used in the preparation of their manuscripts. They must also specify the scope and nature of that use at the time of submission [6,7].

## Harmonizing human judgment and technology

Technological tools should function as supportive instruments that assist, rather than replace, the scholarly judgment of experienced experts. Final verification of research integrity will remain the responsibility of human editors [7].

## Adoption of standardized ethical guidelines

The journal will apply standardized ethical guidelines and actively share patterns of research misconduct by drawing on resources such as the Committee on Publication Ethics (COPE) [8].

## Continuous education

Ongoing education for authors, editors, and reviewers on emerging AI ethics and methods for detecting research misconduct will be essential for strengthening the resilience of the scholarly ecosystem.

## Building a sustainable scholarly ecosystem through collaboration

Safeguarding research integrity cannot be accomplished by individual journals alone; it requires coordinated efforts among researchers, institutions, and publishers at both national and international levels. Beyond merely filtering out problematic manuscripts, the scholarly community must cultivate an environment in which researchers can pursue innovation within a strong ethical framework through education and institutional improvement.

A sustainable research ecosystem also requires changes that extend beyond detection mechanisms alone. These efforts must be accompanied by improvements in incentive structures and academic culture. The prevailing “publish or impoverish (perish)” culture [9], which emphasizes quantitative productivity, can inadvertently encourage misconduct. Accordingly, a shift toward evaluation systems that prioritize research quality and reproducibility is essential.

Innovative uses of AI in research and publishing should be welcomed. However, such innovation must always operate within the fundamental scholarly principle of transparency. WHN remains committed to maintaining a trustworthy and high-quality scholarly record that serves both the academic community and society at large.
